# Predictors of Atrial Fibrillation Recurrence in Hyperthyroid and Euthyroid Patients

**DOI:** 10.5935/abc.20160013

**Published:** 2016-02

**Authors:** Muhammet Gürdoğan, Hasan Ari, Erhan Tenekecioğlu, Selma Arı, Tahsin Bozat, Vedat Koca, Mehmet Melek

**Affiliations:** 1 Edirne State Hospital - Department of Cardiology, Edirne, Turkey; 2 Bursa Postgraduate Hospital - Department of Cardiology, Bursa, Turkey

**Keywords:** Atrial Fibrillation, Recurrence, Arrhythmias, Cardiac, Hyperthyreoidism, Electric Countershock

## Abstract

**Background:**

Atrial fibrillation (AF) is the most common arrhythmia in adults, and is encountered in 10-15% of the patients with hyperthyroidism. Unless euthyroidism is restored, pharmacological or electrical cardioversion is controversial in patients with AF who remain hyperthyroid.

**Objective:**

The aim of this study was to assess the efficacy of electrical cardioversion and predictors of AF recurrence in hyperthyroid and euthyroid patients.

**Methods:**

The study included 33 hyperthyroid (21 males) and 48 euthyroid (17 males) patients with persistent AF. The patients were sedated with intravenous midazolam before undergoing electrical cardioversion delivered by synchronized biphasic shocks. Rates of AF recurrence were recorded.

**Results:**

Mean follow-up was 23.63 ± 3.74 months in the hyperthyroid group and 22.78 ± 3.15 months in the euthyroid group (p = 0.51). AF recurred in 14 (43.8%) and 21 (44.7%) patients in each group, respectively (p = 0.93). Multivariate regression analysis in each group showed that AF duration was the only predictor of AF recurrence, with odds ratios of 1.38 (95% confidence interval [CI] = 1.05 - 1.82, p = 0.02) in the hyperthyroid group and 1.42 (95% CI = 1.05 - 1.91, p= 0.02) in the euthyroid group.

**Conclusion:**

Rates of long-term AF recurrence were similar in successfully cardioverted hyperthyroid and euthyroid patients. The only predictor of AF recurrence in both groups was AF duration.

## Introduction

Atrial fibrillation (AF) is a supraventricular arrhythmia characterized by non-coordinated atrial activation later followed by mechanical dysfunction.^1^ AF is the second most common arrhythmia in cardiology after sinus tachycardia^2^ and affects 10 - 15% of the patients with hyperthyroidism.

Arterial thromboembolism, the most serious complication of AF, frequently results in stroke.^3^ Recent studies have demonstrated that the risk of ischemic stroke increases significantly in patients with AF.^4^ The high incidence of thromboembolic events found in elderly hyperthyroid patients with heart failure and AF is associated with increased mortality and morbidity rates.^5-7^

Since the risk of thromboembolic events is associated with the duration of AF, achieving a sinus rhythm (SR) as early as possible is important to decrease the risk of fatal complications in hyperthyroid patients with AF. Although hyperthyroidism is considered a reversible cause of AF, only two-thirds of the patients return spontaneously to an SR after their thyroid hormone levels return to normal.^8^ For patients who achieve euthyroidism but persist with AF, cardioversion is an option.^6,9^ However, since the risk of thromboembolism is substantial in AF patients, the decision to postpone cardioversion until euthyroidism is restored is controversial.

The aim of this study was to identify predictors of AF recurrence and compare AF recurrence rates in hyperthyroid and euthyroid patients who underwent successful electrical cardioversion.

## Methods

### Study population

Between January 2006 and July 2010, a total of 137 consecutive patients volunteered to participate in this study and underwent elective cardioversion for persistent AF according to clinical indication. Patients with TSH levels below the normal range were considered hyperthyroid. The normal ranges for thyroid hormone levels were 0.34-5.60 *µ*IU/mL for TSH, 2.50-4.20 pg/mL for free T3 (FT3), and 0.58-1.64 pg/dL for free T4 (FT4). Exclusion criteria for participation in the study were severe valvular heart disease, history of previous valvular surgery, severe left ventricular dysfunction (ejection fraction [EF] < 50%), severe left atrial (LA) enlargement (> 5 cm), previous history of electrical or medical cardioversion for AF, history of AF ablation, and paroxysmal AF. After excluding 29 patients with severe valvular heart disease, 21 with left ventricular dysfunction, and six with paroxysmal AF, the final cohort was composed of 81 patients with persistent AF.

The ethics committee of our hospital approved the study protocol, and we obtained informed consent from the entire study cohort. We performed physical examinations of the participants, recorded their use of medications for concomitant systemic diseases, and collected peripheral venous blood samples from them for complete blood count and biochemical analysis.

Patients were defined as hypertensive when presenting a systolic blood pressure (SBP) above 140 mmHg and/or a diastolic blood pressure (DBP) above 90 mmHg on two consecutive measurements with a 6-hour interval, or when using antihypertensive drugs. They were defined as diabetic when having fasting blood glucose levels above 126 mg/dL on two consecutive measurements, or when using oral antidiabetic drugs or insulin.

### Transthoracic echocardiography

All patients underwent transthoracic echocardiography (TTE) before and 24 hours after cardioversion according to the guidelines of the American Society of Echocardiography.^10^ We performed this evaluation with a Vivid7 Pro TTE system and a 3.5 MHz probe with the patient positioned in lateral decubitus. We calculated the left ventricular EF with the Teichholz formula from M-mode images obtained from a parasternal long-axis view and measured the LA volume with the modified biplane area-length method.^11,12^ To calculate the left atrial ejection fraction (LAEF), we used the following formula (in %): (left atrial maximum volume [LAVmax] - left atrial minimum volume [LAVmin]/LAVmaxx100). In the apical four-chamber view, we measured the peak E-wave velocity and E-wave deceleration time with pulsed-wave Doppler (PWD) placing the sample volume (3 mm) between the mitral leaflets tips, and obtained images from the lateral mitral annulus with tissue Doppler imaging (TDI). Gains were minimized, and the TDI filter and Nyquist limits were adjusted to 16 - 20 cm/s to allow for a clear tissue signal. Early diastolic mitral annular velocities (Em) were measured.^13^ All data were averaged from 3 - 5 consecutive beats to deal with AF's variable R-R intervals.

### Transesophageal echocardiography

All patients underwent transesophageal echocardiography (TEE) with a 6-MHz TEE probe to exclude the presence of atrial thrombi before cardioversion. The procedure was performed with continuous one-lead electrocardiographic (ECG) recording and heart rate and blood pressure monitoring. We obtained blood flow velocities (filling and emptying) in the left atrial appendage (LAA) with PWD in the longitudinal view during TEE by positioning the sample volume at the proximal third (about 1 cm inside) of the LAA cavity. The LA and the LAA were carefully scanned in multiple views for evidence of thrombi.

### Cardioversion and follow-up

Anticoagulation was obtained before cardioversion with continuous intravenous infusion of heparin (17 U/kg) in patients with ineffective or without warfarin treatment. The heparin dose was adjusted to maintain the activated partial thromboplastin time (aPTT) at 1.5-2 times the normal value. Euthyroid patients without an intracardiac thrombus in the TTE and TEE examinations were given intravenous amiodarone (loading dose of 5 mg/kg and maintenance dose of 10-15 mg/kg/h for 24 hours). Treatment with a beta-blocker was initiated in patients in the hyperthyroid group as an infusion of esmolol (loading dose of 500 mg/kg for 1 min and maintenance dose of 0.05 mg/kg/min with 0.05 mg/kg/min increments every 5 min according to the ventricular rate to reach a maximum dose of 0.2 mg/kg/min) followed by metoprolol (50-100 mg oral) and propylthiouracil (loading dose of 150-300 mg/day and maintenance dose determined according to the clinical response).

Patients were sedated before cardioversion with intravenous midazolam (initial dose of 3 mg followed by 1 mg injections until sedation). Transthoracic electrical direct current (DC) cardioversion was performed in an intensive care unit with synchronized biphasic DC shocks using a cardioverter-defibrillator (Cardiolife TEC 5531, Nihon Kohden Corporation, Japan). The initial cardioversion energy level was set at 150 J and subsequent levels were 200 J and 270 J. External biphasic DC shocks were applied at the physician's discretion until the highest energy level was reached (270 J) or until SR was restored. The cardioversion was deemed successful when the SR lasted longer than 1 min after the procedure. Patients who restored the SR after cardioversion received effective anticoagulation (international normalized ratio [INR] greater than 2.0) for 1 month with warfarin (initial dose of 5 mg/day later adjusted to maintain the INR at 2 - 3). After discharge from the hospital, amiodarone was interrupted, oral metoprolol 100 - 200 mg/day was maintained, and propafenone 150 - 300 mg/day was initiated (both adjusted according to the heart rate) in both study groups. Heart rate and rhythm were monitored with a 12-lead ECG recording. Patients were evaluated 1, 2, and 4 weeks after the procedure with physical examination, ECG, and an INR measurement. Warfarin was interrupted 1 month after the procedure if the SR was restored and prescribed again according to the thromboembolic risk if the AF recurred. Follow-up visits were performed monthly for heart rhythm monitoring, and patients were advised to seek hospital care immediately if presenting symptoms of palpitations or irregular rhythm.

### Statistical analysis

The data were collected and analyzed with SPSS 10.0 (SPSS Inc, Chicago, IL, USA). Continuous variables were reported as means ± standard deviations and compared with Student's *t* test or Mann-Whitney U test. Categorical variables were expressed as percentages and compared with the chi-square or Fisher's exact test when appropriate. Univariate and multivariate logistic regression analyses were used to determine significant predictors of AF recurrence after cardioversion. The sensitivity and specificity of AF duration to predict AF recurrence were estimated with receiver operating characteristic (ROC) analysis. P values lower than 0.05 were considered statistically significant.

## Results

The cardioversion was successful in 79 patients and unsuccessful in two patients, one in the euthyroid group and the other in the hyperthyroid group. The rate of cardioversion success in the euthyroid and hyperthyroid groups were 97.6% (42 of 43 patients) and 96.9% (32 of 33 patients), respectively. Among patients with successful cardioversion, the male gender prevailed in the hyperthyroid group and the female gender in the euthyroid group (p = 0.006) (Table 1). Diabetes was significantly more frequent in the euthyroid group (p = 0.01) (Table 1). As expected, both groups differed significantly regarding levels of TSH (p < 0.001), FT3 (p = 0.001), and FT4 (p < 0.001) (Table 2). Antithyroid drug treatment was started in hyperthyroid patients soon after establishing the diagnosis of hyperthyroidism and was maintained during cardioversion.

**Table 1:** Baseline characteristics of the patients

	**Hyperthyroid (n = 32)**	**Euthyroid (n = 47)**	**p value**
Age (years)	65.53 ± 6.53	61.17 ± 10.34	0.09
Gender (M/F)	21 (65.6%) / 11 (34.4%)	16 (34.0%) / 31 (66.0%)	0.006
Pulse rate (beats/min)	113.56 ± 18.90	109.63 ± 20.14	0.38
SBP (mmHg)	140.59 ± 16.57	138.93 ± 16.67	0.66
DBP (mmHg)	86.71 ± 9.29	84.78 ± 9.49	0.37
Diabetes mellitus	0 (0%)	8 (17%)	0.01
Hypertension	25 (78.1%)	37 (78.7%)	0.94
Cerebrovascular events	1 (3.1%)	1 (2.1%)	0.78
Coronary artery disease	0 (0%)	2 (4.3%)	0.23
Smoking	5 (15.6%)	4 (8.5%)	0.32
CHA2DS2-VASc score	1.8 ± 1.1	2.1 ± 1.0	0.31
Dyslipidemia	2 (6.7%)	4 (8.5%)	0.71
Aspirin	30 (93.8%)	42 (89.4%)	0.50
Beta-blocker	27 (84.4%)	33 (70.2%)	0.64
CCB	1 (3.1%)	1 (21%)	0.78
ACE inhibitors	24 (75.0%)	33 (70.2%)	0.64
ARB	1 (3.1%)	3 (6.4%)	0.51
Statin	0 (0%)	2 (2.5%)	0.23
Diuretics	0 (0%)	1 (2.1%)	0.40
Warfarin	12 (37.5%)	17 (36.2%)	0.90
AF duration (months)	5.92 ± 4.10	6.22 ± 4.52	0.75
CV energy (J)	214.68 ± 44.72	221.91 ± 43.11	0.47

AF: atrial fibrillation; M: male; F: female; ARB: angiotensin receptor blocker; ACE inhibitors: angiotensin-converting enzyme inhibitors; CCB: calcium channel blocker; CHA2DS2-VASc: scoring system based on the presence of cardiac failure, hypertension, age ≥ 75 years (double weight), diabetes, stroke (double weight), vascular disease, age 65-74 years, and sex category (female); CV: cardioversion; SBP: systolic blood pressure; DBP: diastolic blood pressure.

**Table 2:** Echocardiographic and biochemical findings of hyperthyroid and euthyroid patients

	**Hyperthyroid (n = 32)**	**Euthyroid (n = 47)**	**p value**
Hemoglobin (g/dL)	12.13 ± 1.09	12.49 ± 1.34	0.20
Leukocytes (x10^3^)	8.03 ± 1.43	8.37 ± 1.07	0.22
Platelets (x10^3^)	282.18 ± 67.76	275.65 ± 52.76	0.63
Glucose (mg/dL)	98.93 ± 7.01	105.38 ± 20.84	0.09
Urea (mg/dL)	30.00 ± 8.13	36.80 ± 8.15	0.52
Creatinine (mg/dL)	0.99 ± 0.16	1.00 ± 0.14	0.74
Na (mmol/L)	140.90 ± 2.53	139.82 ± 2.67	0.07
K (mmol/L)	4.55 ± 0.36	4.50 ± 0.35	0.50
TSH (mIU/mL)	0.018 ± 0.003	2.77 ± 1.36	< 0.001
FT3 (pg/mL)	6.67 ± 6.24	2.57 ± 0.60	0.001
FT4 (pg/mL)	2.39 ± 1.23	1.26 ± 0.14	< 0.001
LVEF (%)	60.68 ± 6.72	62.42 ± 6.20	0.24
LA diameter (cm)	4.40 ± 0.36	4.39 ± 0.34	0.89
LA diameter 24^th^ hour (cm)	4.32 ± 0.37	4.36 ± 0.34	0.60
LA maximum volume (mL)	88.11 ± 21.89	79.15 ± 24.07	0.09
LA maximum volume at the 24^th^ hour (mL)	99.76 ± 23.23	92.73 ± 24.33	0.21
LAEF(%)	45.26 ± 5.53	43.78 ± 8.23	0.46
LAEF 24^th^ hour (%)	55.14 ± 4.33	53.96 ± 5.56	0.42
LAAPEV (cm/sec)	0.47 ± 0.07	0.47 ± 0.12	0.92
LAAMEV (cm/sec)	0.39 ± 0.06	0.39 ± 0.10	0.88
LAAPFV (cm/sec)	0.51 ± 0.07	0.50 ± 0.13	0.66
LAAMFV (cm/sec)	0.41 ± 0.07	0.40 ± 0.10	0.54
Mitral E (cm/sec)	0.79 ± 0.14	0.77 ± 0.15	0.54
Mitral E 24^th^ hour (cm/sec)	0.73 ± 0.18	0.69 ± 0.17	0.52
Mitral A 24^th^ hour (cm/sec)	0.47 ± 0.16	0.40 ± 0.16	0.08
Mitral A TVI 24^th^ hour (cm)	9.10 ± 2.73	8.40 ± 2.27	0.48
LV lateral E^1^ (cm/sec)	0.10 ± 0.02	0.10 ± 0.02	0.83
LV lateral E^1^ 24^th^ hour (cm/sec)	0.09 ± 0.03	0.09 ± 0.03	0.77
Mitral E/E^1^	8.17 ± 2.74	8.24 ± 3.47	0.93
Mitral E/E^1^ 24^th^ hour	7.80 ± 2.42	7.90 ± 2.52	0.89

TSH: thyroid-stimulating hormone; FT3: free triiodothyronine; FT4: free thyroxine; LVEF: left ventricular ejection fraction; LA: left atriual; LAEF: left atrial ejection fraction; LAAPEV: LA appendage peak emptying velocity; LAAMEV: LA appendage mean emptying velocity; LAAPFV: LA appendage peak filling velocity; LAAMFV: LA appendage mean filling velocity; E: early diastolic filling wave; A: late diastolic filling wave; TVI: time velocity integral; E^1^: early diastolic tissue Doppler wave.

**Figure 1:**
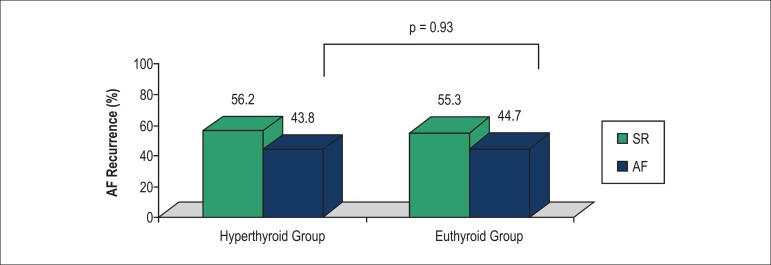
Rates of atrial fibrillation recurrence in hyperthyroid and euthyroid patients. SR: sinus rhythm; AF: atrial fibrillation.

**Figure 2:**
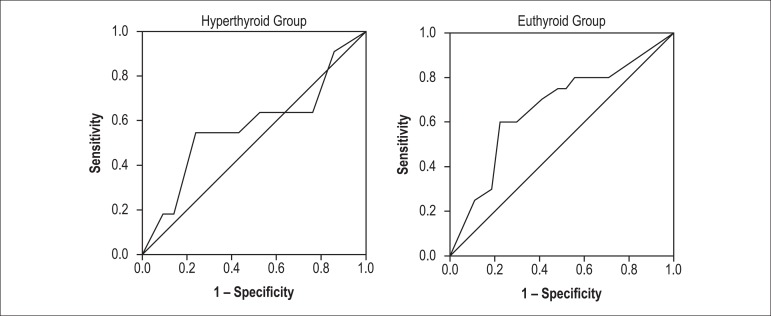
ROC curves showing the sensitivity and specificity rates of AF duration in predicting AF recurrence in hyperthyroid and euthyroid patients.

Patients in the hyperthyroid and euthyroid groups had similar follow-up durations (23.63 ± 3.74 months and 22.78 ± 3.15 months, respectively, p = 0.51), rates of AF recurrence (43.8% [14 patients] and 44.7% [21 patients], respectively, p = 0.93) (Figure 1), and time to AF recurrence (6.81 ± 4.53 months and 7.90 ± 4.22 months, respectively, p = 0.52). In the euthyroid group, univariate regression analysis revealed that age, AF duration, cardioversion energy level, history of hypertension, LAEF, LAA peak emptying velocity (LAAPEV), LAA mean emptying velocity (LAAMEV), LAA peak filling velocity (LAAPFV), and use of angiotensin-converting enzyme (ACE) inhibitors were significant predictors of AF recurrence (p = 0.03, p < 0.01, p = 0.01, p = 0.03, p = 0.04, p = 0.02, p = 0.03, p = 0.048, p = 0.04 respectively). In multivariate regression analysis, AF duration was the only significant predictor of AF recurrence in the euthyroid group (odds ratio [OR] = 1.42, 95% confidence interval [CI] = 1.05 - 1.91), p = 0.02]. In the hyperthyroid group, AF duration (p < 0.01), mitral A-wave velocity at the 24^th^ hour (p = 0.01), and time-velocity integral (p = 0.02) emerged as significant predictors of AF recurrence in univariate regression analysis, whereas AF duration was the only predictor of AF recurrence in multivariate analysis (OR = 1.38, 95% CI = 1.05 - 1.82, p = 0.02). ROC curve analyses showed that the sensitivity and specificity rates of an AF duration of 9.5 months in predicting AF recurrence were 60% and 78%, respectively, in the euthyroid group, and 55% and 76%, respectively, in the hyperthyroid group (Figure 2).

## Discussion

This study evaluated the rates and predictors of AF recurrence after cardioversion in euthyroid and hyperthyroid subjects. The key finding was that long-term AF recurrence rates were similar in hyperthyroid and euthyroid patients after cardioversion and that AF duration was the only parameter predictive of AF recurrence in both groups.

In the clinical setting, AF is the most common rhythm disorder. It is considered an independent risk factor for cardiovascular events^14,15^ and its frequency increases with aging. Age, male gender, ischemic heart disease, congestive heart failure, and heart valve disorders are among the most important risk factors for the development of AF.^15^ Due to rapid and irregular heartbeats, thrombi may form in the heart of patients with AF. Entrance of these thrombi into the bloodstream may cause complications that increase the morbidity and mortality associated with the disease, such as peripheral emboli and stroke in particular.^15^ Chronic AF carries an annual thromboembolic complication risk of 3 - 4%, which is 5 - 7 times higher than that in patients with an SR.^16^

Low serum TSH level is an independent risk factor for AF.^17,18^ A study conducted in more than 23,000 patients has found AF in 2.3% of patients with euthyroidism, 12.7% of those with subclinical hyperthyroidism, and 1 3.8% of those with clinical hyperthyroidism.^19^ Siu et al. have found that during a 1-year follow-up 9.4% of the AF patients with hyperthyroidism have ischemic stroke compared with 3.1% in those without hyperthyroidism.^8^ The incidence of ischemic stroke in hyperthyroid AF patients has been described as significantly higher when compared with that in euthyroid patients.^4,20^

A common treatment approach to AF induced by hyperthyroidism is first to normalize the levels of thyroid hormone.^8^ Even though hyperthyroidism is considered a reversible cause of AF, only 60 - 70% of the hyperthyroid patients return to an SR when the thyroid hormones are normalized, with the remaining 30 - 40% maintaining permanent AF.^8^ The return to an SR occurs within the first 8 - 10 weeks after the levels of thyroid hormones return to normal.^21^ This is a very long period considering the impact of a thromboembolic complication.^9^ Restoration of an SR decreases thromboembolic risks and improves cardiac pump function.^8^

Recurrence of AF affects more than one-third of the patients in the first two weeks after cardioversion. The risk of recurrence decreases later on and becomes stable during the follow-up period.^22^ A study has reported that while the cardioversion success rate is around 90% in cases with AF duration shorter than 1 year, the recurrence risk is 40% in the first 6 months and 50 - 60% at the end of the first year, even when antiarrhythmic medications are used.^23^ The mean AF duration in our patients was 6 months and 97.5% (79 out of 81) achieved an SR. This high success rate in our patients may be explained by their short AF duration, small LA, premedication with antiarrhythmic drugs, young age, and adequate left ventricular function. In patients with hyperthyroidism, the hyperthyroid state preserved the LA and LAA contractile functions, which increased the cardioversion success rate.

A study conducted by Emery and Staffurth^24^ has demonstrated that following a successful cardioversion, 45% of the patients maintain an SR during 2 years. This ratio decreased to 36% when the follow-up was longer (mean 7.4 years).^24^ Siu et al.^8^ have shown that AF recurrence rates 24 months after a successful cardioversion were 59% in patients with hyperthyroidism compared with 83% in those without thyroid dysfunction.^8^ The risk of AF recurrence in our study was similar in patients with hyperthyroidism (14 patients, recurrence rate 43.8%) and euthyroidism (21 patients, recurrence rate 44.7%, p = 0.93). The different recurrence rates in our study compared with others may be due to different AF durations and echocardiographic features.^21-24^ A similar study has shown a risk of recurrence of 30% in the first year after cardioversion, which increased to 60 and 79% when the cardioversion was postponed to 12 and 36 months, respectively.^8^ The risk of AF recurrence was lower in patients with hyperthyroidism when compared with those with euthyroidism,^8^ although another study similar to ours found comparable rates in both groups.^25^ Considering the complications of AF and the long 8- to 10-week period required for thyroid hormone levels to normalize, cardioversion can be performed in hyperthyroid patients even before achievement of euthyroidism.

The main predictors of AF recurrence after successful cardioversion are severe left ventricular dysfunction, LA enlargement, and long duration of the previous AF.^22^ Long AF duration leads to atrial enlargement and development of more reentrant atrial circuits. Also, fibrous and inflammatory changes in the atrial myocardial tissue shorten the atrial conduction time. These factors lead to permanent AF and higher recurrence rates.^26,27^ In our study, predictors of AF recurrence were evaluated both in hyperthyroid and euthyroid subjects and the only significant predictor of AF recurrence was long AF duration.

Increased levels of C-reactive protein (CRP), atrial natriuretic peptide (ANP), and brain natriuretic peptide (BNP), and decreased levels of aldosterone have been shown to be predictors of AF recurrence. These biochemical markers reflect inflammation, neurohormonal activation, and activation of the renin-angiotensin-aldosterone system. There is growing interest in exploring the participation of inflammatory and oxidative stress in the pathophysiology of AF. A meta-analysis has suggested that increased baseline CRP levels were associated with a higher risk of AF recurrence after successful electrical cardioversion, although there was significant heterogeneity among the studies.^28^ Both ANP and BNP levels increase in patients with AF due to neurohormonal activation and decrease after successful cardioversion. However, studies evaluating the predictive value of natriuretic peptides is conflicting.^29^ Recently, other pathophysiologic factors associated with the renin-angiotensin-aldosterone system were evaluated through determination of aldosterone levels. Serum aldosterone level is a marker of atrial structural remodeling, and lower levels have been associated with a lower rate of AF recurrence.^30^ Although we did not evaluate biochemical markers in our study, hyperthyroidism may influence the ANP and BNP levels, which may have interfered with the results.

In euthyroid patients, an electromechanical delay in the LA and the P-wave duration have been shown to be predictors of AF recurrence.^31,32^ Also, studies assessing P-wave duration have shown significant heterogeneity.^32,33^

### Study limitations

The main limitation of our study is its small sample size. Despite of that, our study presents an alternative treatment for hyperthyroid patients with AF that may prevent fatal complications. Another limitation is that we did not evaluate biochemical markers, or electromechanical and electrocardiographic parameters, which could be affected by many factors. The preliminary findings of the present study should be confirmed in controlled clinical studies with a larger sample size of patients with AF induced by hyperthyroidism.

### Clinical implication

Hyperthyroidism is a cause of AF, and only 65 - 70% of the patients return spontaneously to an SR after achieving normal hormone levels. All patients with AF regardless of having hyperthyroidism or euthyroidism have a substantial risk of thromboembolism. To prevent this complication, hyperthyroid patients may undergo cardioversion even before achieving euthyroidism.

## Conclusion

Rates of AF recurrence are similar in hyperthyroid and euthyroid patients, and the duration of AF is the only predictor of AF recurrence in both.
